# Can implementation support help community-based settings better deliver evidence-based sexual health promotion programs? A randomized trial of Getting To Outcomes®

**DOI:** 10.1186/s13012-016-0446-y

**Published:** 2016-05-31

**Authors:** Matthew Chinman, Joie Acosta, Patricia Ebener, Patrick S. Malone, Mary E. Slaughter

**Affiliations:** RAND Corporation, 4570 Fifth Avenue, Pittsburgh, PA 15213 USA

**Keywords:** Implementation support, Fidelity, Evidence-based prevention, Community-based

## Abstract

**Background:**

Research is needed to evaluate the impact of implementation support interventions over and above typical efforts by community settings to deploy evidence-based prevention programs.

**Methods:**

Enhancing Quality Interventions Promoting Healthy Sexuality is a randomized controlled trial testing Getting To Outcomes (GTO), a 2-year implementation support intervention. It compares 16 Boys and Girls Club sites implementing Making Proud Choices (MPC, control group), a structured teen pregnancy prevention evidence-based program with 16 similar sites implementing MPC augmented with GTO (intervention group). All sites received training and manuals typical for MPC. GTO has its own manuals, training, and onsite technical assistance (TA) to help practitioners complete key programming practices specified by GTO. During the first year, TA providers helped the intervention group adopt, plan, and deliver MPC. This group then received training on the evaluation and quality improvement steps of GTO, including feedback reports summarizing their data, which were used in a TA-facilitated quality improvement process that yielded revised plans for the second MPC implementation. This paper presents results regarding GTO’s impact on performance of the sites (i.e., how well key programming practices were carried out), fidelity of MPC implementation, and the relationship between amount of TA support, performance, and fidelity. Performance was measured using ratings made from a standardized, structured interview conducted with participating staff at all 32 Boys and Girls Clubs sites after the first and second years of MPC implementation. Multiple elements of fidelity (adherence, classroom delivery, dosage) were assessed at all sites by observer ratings and attendance logs.

**Results:**

After 2 years, the intervention sites had higher ratings of performance, adherence, and classroom delivery (dosage remained similar). Higher performance predicted greater adherence in both years.

**Conclusions:**

These findings suggest that in typical community-based settings, manuals and training common to structured EBPs may be sufficient to yield low levels of performance and moderate levels of fidelity but that systematic implementation support is needed to achieve high levels of performance and fidelity.

**Trial registration:**

ClinicalTrials.gov, NCT01818791

## Background

Many evidence-based prevention programs or practices (EBPs) are not achieving expected outcomes in typical community settings, raising doubts about the quality of their implementation [1]. Implementation support interventions that can facilitate the successful delivery of EBPs are becoming available. Yet, there is little theory-driven research using rigorous designs that test whether these support interventions improve program delivery or participant outcomes. This is especially the case in the domain of teen pregnancy and sexually transmitted infection (STI) prevention, where programs are often implemented in low-resource, community-based settings that some argue have been under-studied in implementation science [2]. Enhancing Quality Interventions Promoting Healthy Sexuality (EQUIPS) is a 2-year, randomized controlled trial of an implementation support intervention called Getting To Outcomes® (GTO). EQUIPS tested GTO’s impact in helping a community-based setting (Boys and Girls Club sites) implement an evidence-based, teen pregnancy and STI prevention program called Making Proud Choices (MPC) [3]. This paper aims to answer the questions:

1. How much GTO support did sites receive?
2. After 2 years, what is the impact of GTO on sites performance (i.e., whether key programming practices were carried out) and their fidelity of MPC implementation?
3. Is there empirical support for the GTO logic model (the underlying theory that GTO implementation support predicts performance and, consequently, fidelity)?

### Difficulty implementing evidence-based teen pregnancy and STI prevention programs

Although teen pregnancy rates have been declining recently, teen pregnancy and STI continue to be problematic for the USA. In 2013, there were almost 27 births per 1000 adolescent females ages 15–19 (274,641 babies), 89 % of which were outside of marriage [4]. Sexually active teens are at high risk for contracting STIs and other poor outcomes (e.g., dropping out of school, requiring public assistance, living in poverty) [5–7]. These outcomes cost the USA between $9.4 and $28 billion a year from public assistance expenditures, uncollected tax revenue, and public health, foster care, and criminal justice costs [4, 8].

These poor outcomes highlight the need for implementation support in community-based settings. The US Department of Health and Human Services has identified 35 EBPs that have reduced rates of teen pregnancy and STI [9]. Yet communities often face difficulty implementing these EBPs with the quality needed to achieve outcomes demonstrated by researchers [10, 11], yielding a “gap” between research and community practice. This gap [1, 12] often results from limited resources and a lack of capacity—the knowledge, attitudes, and skills—individual practitioners need to implement “off the shelf” EBPs.

### Getting To Outcomes—an implementation support intervention

Getting To Outcomes (GTO) builds capacity for implementing EBPs by strengthening the capacity (i.e., knowledge, attitudes, and skills) needed to carry out practices which are critical to running any program successfully [13], namely goal setting, planning, process and outcome evaluation, and using data to improve and sustain programs. GTO builds capacity through three types of assistance: (1) the GTO manual of text and tools originally published by the RAND Corporation [14] and then adapted for teen pregnancy and STI prevention by the Centers for Disease Control and Prevention [15], (2) face-to-face training, and (3) ongoing, onsite technical assistance (TA). Important to GTO’s capacity-building is asking practitioners to be active learners, setting the expectation, and giving them the opportunity to carry out for themselves the key programming practices GTO specifies. Although EQUIPS applies GTO to teen pregnancy and STI prevention programs, GTO is a generic set of supports that is able to build capacity for any type of program. For example, GTO has been applied to drug use prevention [14], underage drinking prevention [16], and positive youth development [17].

**Fig. 1:**
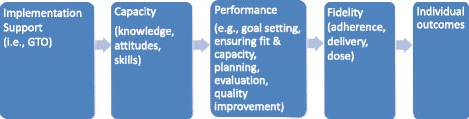
Implementation support logic model

The GTO logic model shows the proposed mechanisms through which GTO works [18] (Fig. 1). The logic model was initially developed based on our observations of how community-based groups needed capacity-building to successfully carry out substance abuse prevention programming [19] and then has been refined based on the research and theories described below. It begins with an implementation support intervention (i.e., GTO) designed to build capacity (i.e., knowledge, attitudes, and skills) to carry out a range of programming practices needed to implement an EBP mentioned above (beyond just program delivery). We define *performance* as the level of quality at which these practices are carried out. Consistent with social cognitive theories of behavioral change [20–23] and implementation science theories such as the Consolidated Framework For Implementation Research (for details, see Acosta et al. and Smelson et al. [24, 25]), we theorize that exposure to GTO through training and TA leads to more capacity to perform these practices, which in turn can improve the performance of the program [26]. Improved performance, in turn, improves program implementation, such as demonstrating program fidelity. EBPs delivered with high fidelity tend to produce positive outcomes [26].

### Previous efforts to evaluate implementation support

The Centers for Disease Control and Prevention and the US Department of Health and Human Services have used implementation support interventions to help community-based organizations adopt and implement EBPs to prevent teen pregnancies and STIs. However, these efforts were not evaluated using rigorous research designs [10, 11, 27–29]. Research has evaluated implementation support interventions in other areas conducted in low-resourced, community-based settings. For example, in substance abuse prevention, the Communities That Care [30] and Promoting School-Community-University Partnerships to Enhance Resilience [31] interventions have shown improvements in EBP fidelity and outcomes in multi-site trials [30, 31]. However, neither tracked what programming was being implemented, or its fidelity, in the control communities. Rohrbach et al. [32] found that standard training plus implementation support (i.e., TA) yielded better fidelity to a substance abuse prevention EBP than standard training alone. That trial was not able to track TA usage or blind fidelity observers, however. With practitioners of drug prevention programs, GTO has been found to improve the capacity of individual practitioners and the performance of prevention programs in both quasi-experimental [33] and randomized controlled trials [24, 34]. However, those studies involved mostly non-evidence-based programs of widely varying type and quality.

### Contributions of the EQUIPS study

EQUIPS builds upon previous governmental initiatives and implementation support research to date in two important ways. First, the design isolates the impacts of GTO by having both the experimental and control conditions receive training in the same EBP, while providing GTO only to experimental sites. In all four previous research studies on GTO, GTO was used at sites that had all different prevention programs [24, 33–36]. Therefore, those studies were limited to a generic measure of program performance that could be compared across different programs. Although we use that measure of performance in EQUIPS, the use of a single EBP allows us to go further and compare the experimental and control conditions with the same fidelity measure. Second, EQUIPS further empirically tests the GTO logic model’s [3, 18] linkages from implementation support (i.e., GTO), to program performance and then to program fidelity, all in a single randomized controlled trial of a teen pregnancy and STI EBP. To our knowledge, there have been no randomized controlled trials that assess implementation support interventions in teen pregnancy and STI prevention.

## Methods

### Design overview

EQUIPS is a 2-year randomized controlled trial (RCT) comparing 16 Boys and Girls Clubs (BGCs) who received typical training to implement the Making Proud Choices (MPC) program (control group) [37] with 16 BGCs who received the same MPC training, plus GTO tools, training, and TA (intervention group). We collaboratively decided upon MPC with the BGCs because of their need for teen pregnancy and STI prevention and MPC’s evidence base and culturally appropriate curriculum [37]. GTO is provided over a 2-year period, allowing all sites to deliver MPC twice. The trial assessed three sets of variables: quality of performance in carrying out key programming practices (e.g., goal setting, planning, evaluation), fidelity of MPC (e.g., adherence, classroom delivery, dosage), and the sexual health outcomes of participating middle school youth. In this paper, we report on GTO’s impact on performance and fidelity and whether there is a relationship between amount of implementation support, performance, and fidelity. We chose these measures because they align with the GTO logic model, which specifies that implementation support improves performance, which in turn improves fidelity.

### Study sites

Based on available study resources, power calculations showed that the study could accommodate *N* = 32 sites. Our power calculation for program performance was conducted under the assumptions, based on previous GTO research [33], that the within-program correlation of scores between baseline and follow-up would be 0.5, while the score in an individual program’s follow-up measurements will be 0.7. Thus, with 32 sites, we calculated that there would be 80 % power to detect medium-to-large effects (effect size = 0.7) in scores over time for programs in the intervention vs. control groups (alpha = 0.05), which is consistent with effects found in GTO studies [24, 33–35]. Power for fidelity was calculated to be similar, especially given that large differences in curriculum adherence rates have been found between researcher- [37] and community-conducted [38] studies.

EQUIPS involves 32 BGC sites in Atlanta, Georgia (*n* = 16) and multiple locations in Alabama (*n* = 16). These sites were chosen because teen pregnancy rates are generally higher in the Southeastern US and they had access to large numbers of youth. In Atlanta, the BGC club offered 16 sites out of 26 based on their demographics of those sites (i.e., those with sufficient numbers of middle school aged youth who could participate). In Alabama, all three BGCs we approached agreed to participate and again offered a combined 16 sites that had sufficient numbers of appropriate youth. BGCs provide youth programming that ranges from recreation in large common rooms and gyms to leadership, character education, health and wellness, and academic programs. A BGC is often made up of several sites (i.e., geographic locations). Although there is some variability across sites, each site has its own facility and a small number of staff and part-time volunteers (*n* = 7–10).

Two to three staff from each site participated and provided consent for all site-level measures. The sites had similar staff demographics. The Alabama sites as a whole, and in the control and intervention groups, had largely similar demographic makeup (no significant differences). Two thirds of the staff were female; most were aged 50–65 (50 %) or 26–49 (44 %); most (88 %) had some college education or greater; and 81 % were African-American and 19 % were White. As a whole, 68 % of the Georgia staff were female; most were aged 50–65 (50 %) or 26–49 (50 %); 100 % had some college or greater education; and 81 % were African-American and 19 % were White. There were no significant differences between the control and intervention groups in Georgia on gender, education, or race; however, the intervention sites’ staff were somewhat older (89 % were 50–65 vs. 17 %).

Using a random number generator, we randomized at the BGC site level, stratified by state, so each state had eight control and eight intervention sites, for a total of 16 sites in both the control and intervention groups. After randomization, the principal investigator of the study informed each site about which group they had been assigned.

At baseline (after randomization), we conducted a web-based survey of staff scheduled to plan and deliver MPC to assess pre-existing variation that might affect the implementation of MPC or the use of GTO. We measured individual capacity for quality prevention (coefficient alpha = 0.83 for knowledge scale, 0.94 for skills scale) [24], attitudes toward EBPs (coefficient alpha = 0.71 to 0.93 across four scales) [39], and organizational support for EBPs (coefficient alpha = 0.65) [40]. Response rates were 71 % (17/24) and 100 % (19/19) for the intervention and control groups, respectively. We evaluated group differences on each scale by fitting a site-level linear mixed effects regression model with fixed treatment (intervention vs. control) effect and a random state (Alabama, Georgia) effect and found no significant differences between the two groups at baseline, although these analyses were only powered to detect medium-to-large effects.

### Making proud choices—an evidence-based pregnancy and STI prevention program

Making Proud Choices (MPC) uses social cognitive theory [41] and the theories of reasoned action [23] and planned behavior [42] to influence adolescents’ knowledge and beliefs about sex and contraception to reduce the frequency of sexual activities and to increase condom use [37, 43]. Over eight, 1-h highly scripted class sessions called “modules,” MPC (1) provides information about abstinence, pregnancy and safe-sex (i.e., condoms); (2) strengthens attitudes of sexual responsibility and pride needed for abstinence and/or safer-sex decision making (e.g., condom use); and (3) teaches skills on how to remain abstinent or use safe-sex practices. The evidence for MPC comes from a rigorous RCT involving primarily African-American sixth and seventh graders from three middle schools serving low-income communities in Philadelphia where half of the youth received MPC and half did not. Immediately at post intervention, the youth that received MPC significantly improved, compared to the control youth, on eight of the 14 mediator measures (i.e., proximal outcome), mostly involving condom use and sexual knowledge. The youth that received MPC also had significantly higher frequency of condom use at 3, 6, and 12 month follow-ups compared to the control youth and significantly less frequent sex and unprotected sex at 6 and 12 months among those who were sexually active at the start of the study [37]. MPC is one of the most popular teen pregnancy and STI prevention programs in the USA. Grantees in the Administration for Children and Families’ Personal Responsibility Education Program (formula state funding for evidence-based teen pregnancy prevention) are using MPC in 18 of the 45 participating states, reaching nearly 64,000 youth [44].

### Making proud choices implementation supported by GTO

Using existing staff, each BGC site was asked to implement MPC once a year for 2 years with a different group of youth each year, staggered across a 3-year timespan from 2012 to 2014. Two half-time TA providers (one in Atlanta, one in Alabama) delivered standard MPC manuals and training to all sites. These providers also provided the intervention group with GTO manuals of text and tools mentioned above, face-to-face training, and onsite TA for 2 years. The GTO manual contains written guidance about how to complete ten GTO steps, with each step being a different set of prevention practices important to successfully carrying out an EBP. Most GTO steps contain “tools,” worksheets that prompt practitioners to make, and then record, decisions about various practices. For example, the GTO goals tool has prompts that assist in the writing of goal and outcome statements. Table 1 shows how BGC staff performed the various prevention practices in each of the ten steps to implement MPC.

**Table 1:** Manual information and practices performed by BGC club staff by each of the 10 GTO steps

GTO step	What the GTO manual provides for each step	Practices BGC club staff carried within each GTO step
1. Needs: What are the needs to address and the resources that can be used?	Information about how to conduct a needs and resources assessment	Club leaders reviewed data about the needs of a their membership
2. Goals and outcomes: What are the goals and desired outcomes?	Tools for creating measurable goals and desired outcomes	Each site developed their own broad goals and “desired outcomes”—statements that specify the amount and timing of change expected on specific measures of knowledge, attitudes, behavior
3. Best practices: Which evidence-based programs can be useful in reaching the goals?	Overview of the importance of using evidence-based programs and where to access information about them	Club leaders reviewed options and choose Making Proud Choices as the evidence-based program to implement
4. Fit: What actions need to be taken so the selected program fits the community context?	Tools to help program staff identify opportunities to reduce duplication and facilitate collaboration with other programs.	Each site reviewed Making Proud Choices for how it would fit within their club and made adaptations to improve fit
5. Capacity: What capacity is needed for the program?	Assessment tools to help program staff ensure there is sufficient organizational, human and fiscal capacity to conduct the program	Each site assessed their own capacity to carry out Making Proud Choices and made plans to increase capacity when needed
6. Plan: What is the plan for this program?	Information and tools to plan program activities in detail	Each site conducted concrete planning for doing Making Proud Choices (e.g., who, what, where, when)
7. Process evaluation: How will the program implementation be assessed?	Information and tools to help program staff plan and implement a process evaluation	Each site collected data on fidelity, attendance, satisfaction to assess program delivery and reviewed that data immediately after implementation
8. Outcome evaluation: How well did the program work?	Information and tools to help program staff implement an outcome evaluation	Each site collected participant outcome data on actual behavior as well as on mediators such as attitudes and intentions
9. Continuous quality improvement: How will continuous quality improvement strategies be used to improve the program?	Tools to prompt program staff to reassess GTO steps 1–8 to stimulate program improvement plans	Each site reviewed decisions made and tools completed before implementation and data collected during and after implementation and made concrete changes for the next implementation
10. Sustainability: If the program is successful, how will it be sustained?	Ideas to use when attempting to sustain an effective program	Each site took steps such as securing adequate funding, staffing, and buy-in, to make it more likely that Making Proud Choices would be sustained

**Fig. 2:**
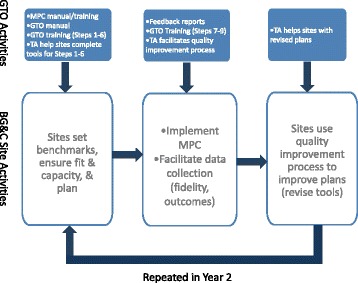
Getting To Outcomes flow

Leading up to the intervention group’s first MPC implementation (Fig. 2), TA providers delivered GTO training to participating BGC staff in multiple sessions, addressing two GTO steps per session up through step 6 (planning). Simultaneously, TA providers helped BGC staff complete each GTO step (e.g., completing tools) to guide the planning of the MPC program during bi-weekly meetings. Then BGC staff implemented MPC and facilitated the collection of fidelity and youth outcome data (described below). BGC sites then received training on evaluation and quality improvement (GTO steps 7–9), along with feedback reports summarizing evaluation data from their sites, which were used in a TA-facilitated quality improvement process that resulted in a revised plan for the second implementation of MPC. The 2-year implementation followed the same process and collected the same data, supplemented by training on sustainability (GTO step 10). All BGC sites received $3000 a year to defray some costs of participating in the study. Chinman et al. [45] provides additional details about the use of GTO with MPC.

### Measures and data collection

EQUIPS was approved by RAND’s Institutional Review Board. Harms of GTO and MPC were monitored by data collectors and TA staff during the 3-year timespan GTO was active. None were reported.

#### Amount of GTO implementation support

TA providers recorded the hours of training and TA they delivered to each site, by GTO step, on the *Technical Assistance Monitoring Form*. Hours of support have shown to be related to our measure of performance (described below) in previous studies of GTO [33, 35]. Time spent training on MPC was recorded under GTO step 3.

#### Program performance

As in past GTO studies [33, 34], we used the structured *Program Performance Interview* with the staff member most responsible for MPC at each site. Although programs consist of individual people with varying abilities, ratings are made at the site level because programs operate as a unit. Each site was assessed twice by one of two interviewers. In the intervention group, the interviews were conducted each year after staff reviewed their evaluation data and made a quality improvement plan. In the control group, the interviews were conducted each year one month after MPC ended.

The interview consists of 12 items that assess how well practices in eight domains key to program success (i.e., that align with eight GTO steps) are performed throughout MPC implementation: developing goals and desired outcomes, ensuring program fit, ensuring sufficient capacity, planning, process evaluation, outcome evaluation, continuous quality improvement, and sustainability. Each item is rated on a five-point scale from “highly faithful” to ideal practice (5) to “highly divergent” from ideal practice (1). All the items have specific criteria that guide the ratings. The measure yields a score for each domain and a total score. Because we and the clubs’ leaders jointly agreed to use MPC prior to the study, we did not assess practices related to needs assessment (step 1) or selecting a best practice (step 3) because these activities were not required of the individual sites.

All interviews were audio recorded. In year 1, 13 % of interviews were rated by both interviewers to calculate inter-rater reliability (intra-class correlation or ICC, across all scores = 0.74). In year 2, 13 % were also double coded and ICC across all scores was 0.30. However, we also calculated percent agreement in year 2 given the sample size and range of scores were limited for calculating ICC with precision. Across all individual scores in year 2, 78 % were an exact match between the two coders and an additional 18 % were one point off. It was not possible to blind these interviews because intervention respondents talked explicitly about GTO activities. This measure has been sensitive to change and reliable in previous GTO studies [33, 34].

#### MPC fidelity

All sites were rated on three dimensions of fidelity—adherence, quality of delivery, and dosage [46]. Ratings were made by research data collectors (blind to condition), who received 6 h of initial training on the MPC fidelity protocol, attended weekly supervisory meetings, and participated in quarterly refresher trainings.

##### Adherence

Data collectors observed and rated two to three MPC modules per site (randomly selected) on how closely BGC staff implemented the activities in the module as designed (not at all, partially, fully) using an MPC fidelity tool [47]. In each year, a total of 1472 activities were conducted across all 32 sites (a full MPC program contains 46 discrete activities). In year 1, we observed and rated 537 of those activities (36 %), distributed across all 32 sites (*n* = 260 for the control group, 289 for the intervention group). In year 2, we observed and rated 303 activities (21 %), distributed across all 32 sites (*n* = 134 for the control group, 169 for the intervention group). Across all double coded adherence scores, Cohen’s weighted Kappa was 0.92 in year 1 and 0.96 in year 2.

##### Quality of MPC delivery

At the same visits, data collectors rated BGC staff on level of facilitator’s classroom control, level of facilitator enthusiasm, degree to which the facilitator met objectives of the module, and student interest—all on a 1–7 scale (7 = most control/enthusiasm/objectives met/interest). We double coded 5 % of these observations to calculate inter-rater reliability. ICCs for the four qualities of delivery scores ranged from 0.48 to 0.70 in year 1 and 0.43 to 1.0 in year 2.

##### Dosage

BGC staff at control and intervention sites recorded the attendance of the enrolled youth at each MPC module and transmitted the counts to RAND.

### Analyses

#### Overview

The experimental unit for all analyses was *site*, which was the level randomized into the two treatment groups. We compared intervention group sites with control sites on three measures: GTO implementation support (TA hours); program performance; and MPC fidelity (adherence, delivery, and dosage). The observational unit was *site* for analyses involving TA hours, program performance, and delivery, *MPC activity* for adherence analyses, and *student* for dosage analyses. We fit separate models for years 1 and 2. We also assessed changes in outcome for each study group from years 1 to 2 by fitting a model including both years and testing the effect of year in the intervention and control groups. The null hypotheses in these tests were that the outcome was the same for both years, i.e., no year-to-year change within group. We also tested whether the change from year 1 to 2 for the intervention group differed from the change from year 1 to 2 in the control group, a test of the moderating influence of year of implementation. All analyses were conducted in SAS v9.4, predominantly with PROC MIXED and PROC GLIMMIX. All the analyses are summarized, along with their results, in Table 5.

#### Amount of GTO implementation support

Means and standard deviations were calculated for total TA hours and by GTO step. Given that past GTO studies experienced significant intervention bleed into control groups [24, 33], we conducted multiple comparisons of TA hours by treatment group for (1) the GTO steps in which the control group had any hours (steps 3–4, 6, and 7 in year 1 and steps 1 to 9 in year 2) and (2) total TA hours. For each comparison, we fit a linear mixed effects regression model with fixed treatment effect (control vs. intervention group) and a random state effect (Alabama, Georgia). We used one-parameter *t* tests of the coefficient associated with treatment assignment to evaluate differences between means of a continuous outcome stemming from a two-group fixed effect, while considering the degrees of freedom. We report the treatment effect, *M*_diff_, 95 % confidence interval for the treatment effect, *t* statistic, degrees of freedom, and a post hoc adjusted *p* value. Effect sizes for two-mean comparisons (i.e., Hedges’ *g*) were calculated by dividing the treatment effect by the square root of the mean squared error.

For the interaction terms in the change between years analysis, we report generalized omega-squared as the estimate of effect size, calculated using a SAS macro designed by Kellerman et al. [48] We modified the macro to use an effective *N*, where effective *N* = *N*_(total sample)_/design effect and design effect = 1 + ICC (*n* − 1), to account for the random effects in the models. Omega-squared, like eta-squared, represents an estimated proportion of variance accounted for the term in the linear model, but is minimally biased relative to eta-squared’s known inflation and is the variation least sensitive to design characteristics. Unfortunately, appropriate confidence intervals for omega-squared are not yet well developed, so we report only the point estimate. Confidence intervals *are* known to tend to be larger than for other measures of proportion of variance. The negative point estimates for omega-squared in some of our results reflect that uncertainty [49].

#### Program performance

Means and standard deviations were calculated by GTO step (*n* = 8) and for a total score. We compared control and intervention groups by again fitting a site-level linear mixed effects regression model similar to the TA hours analysis described above, using performance scores for each GTO step and the total score as the dependent variables. Tests of the treatment effects within and across years are reported similarly to the TA hours analysis.

#### MPC fidelity

We compared control and intervention groups across all three dimensions of fidelity. For adherence, we fit a mixed effects logistic regression model similar to the models for TA hours and program performance, except in this analysis, we used site-level random effects nested within state since the observational unit was one rated MPC activity and we wished to account for possible correlation between activities within a site. Treatment was again the only fixed effect. Adherence was treated dichotomously to isolate the effect of GTO on the two ends of the fidelity spectrum (not at all vs. full). We compared the rating of “not at all” vs. [“partially” + “fully”] and [“not at all” + “partially”] vs. “fully.” However, in year 2, we were only able to compare [“not at all” + “partially”] vs. “fully” dichotomization because of small cell sizes for the “not at all” ratings. The model was fit by maximizing the residual log pseudo-likelihood and type III tests were used to determine significance of the treatment effect. We report odds ratios and 95 % confidence intervals for the treatment effect for the separate year 1 and year 2 analyses, and for the change from year 1 to year 2 by group, and logistic regression coefficient and CI for tests of moderation in the combined years analyses.

For quality of delivery, we used similar, site-level models to those used for program performance and TA hours. In these models, the outcome was the raw 1 to 7 scale rating, treated as a continuous variable in a linear mixed effects regression model. The four models (classroom control, facilitator enthusiasm, objectives met, and student interest) were fit using restricted maximum likelihood.

Finally, for dosage, we compared respondents’ attendance from the control and intervention groups using a linear mixed effects model where the outcome was percent modules attended (out of eight), the fixed effect was treatment group, and the random effects were intercepts for sites within states. We tested treatment differences in each year and between years with the approach used for the TA hours and program performance analyses.

#### Support for GTO logic model

We conducted analyses to further examine the relationship between implementation support (i.e., GTO TA hours), program performance, and fidelity, consistent with the GTO logic model described above [3, 18]. First, we used TA hours to predict program performance in intervention sites using a linear mixed effects regression model with TA hours as the fixed effect and site-level intercepts within state as the random effect. We fit a separate model for TA hours spent on each of the eight GTO steps and for total hours. We restricted the analyses to intervention sites because only those sites were intended to receive TA hours and the number of sites was not sufficient to test condition moderation of the effects of TA hours. Next, we examined whether average program performance scores predicted adherence across all sites. To do this, we fit a mixed effects logistic regression model at the activity level similar to the models used in the analysis of the adherence ratings, except here we controlled for a fixed effect of site-level average program performance score.

#### Type I error control

Due to the multiple contrasts in many of the outcome analyses, we adjusted *p* values using the Benjamini-Hochberg procedure [50] to control the false discovery rate (FDR) across the tests. A false discovery correction is designed to adjust *p* values such that, across significant findings after adjustment, a proportion of approximately α (0.05 herein) will reflect type I error. We made this correction within three sets of multiple tests addressing the same conceptual result: the tests of TA hours in each GTO step; the tests of GTO performance scores in each GTO step; and the tests of four MPC classroom context scores. We made the corrections separately within the analyses for year 1 and year 2 and the difference between years because these analyses address different conceptual questions.

## Results

### Amount of GTO implementation support

As in past GTO studies [24, 33], the control group did inadvertently receive some TA hours, not including the time devoted to MPC training (recorded under step 3). However, the mixed effects regression models for TA hours showed intervention sites received significantly more TA hours compared to the control group for all models in both years even after FDR adjustment (see Table 2 for details, Table 5 for a summary). Specifically in year 1, the intervention sites had more hours in GTO steps 3, 4, 6, and 7 (steps in which TA hours were recorded for both groups) and total hours than control sites. Including the time it took to deliver the MPC training, intervention sites received about a total 35 h per site compared to about 8 h in the control group. Subtracting the MPC training hours out, it was 21 and 4 h for the intervention and control sites, respectively.

**Table 2:** TA and training hours in years 1 and 2

GTO steps	Year 1^b^			Year 2^b^			Change from year 1 to year 2		
	M(SD)		Hedges’ g (95 % CI)	M(SD)^b^		Hedges’ g (95 % CI)	Hedges’ g (95 % CI)		Generalized omega-squared^c^
	Control	Intervention		Control	Intervention		Control	Intervention	Difference of differences
1. Needs assessment	0	1.0(0.8)	NA	0.1(0.1)	0.52(0.8)*	0.87 (0.12, 1.61)	NA	NA	NA
2. Goals	0	0.9(0.7)	NA	0.1(0.2)	0.89(0.8)**	1.43 (0.63, 2.23)	NA	NA	NA
3. Best practices^a^	4.5(7.6)	13.8(7.2)**	1.25 (0.48, 2.02)	7.8 (10.7)	20.2(19.6)*	0.88 (0.14, 1.63)	0.35 (–0.36, 1.06)	0.46 (–0.27, 1.18)	−0.019
4. Fit	1.4(1.3)	3.4(1.7)***	1.87 (1.02, 2.71)	0.7 (1.2)	2.1(1.8)*	1.03 (0.27, 1.79)	−0.87 (−1.61, −0.14)	−1.48 (−2.29, −0.67)***	0.004
5. Capacity	0	3.7(4.6)	NA	0.1 (0.2)	3.7(4.2)**	1.18 (0.41, 1.96)	NA	NA	NA
6. Planning	2.2(2.0)	6.2(2.6)***	1.87 (1.02, 2.71)	1.2 (1.3)	6.8(4.4)***	1.80 (0.97, 2.62)	−1.19 (−1.96, −0.43)	0.17 (−0.54, 0.89)	0.011
7. Process evaluation	0.1(0.1)	0.7(0.7)**	1.27 (0.50, 2.04)	0.1(0.3)	1.3(1.7)*	0.92 (0.16, 1.67)	0.90 (0.17, 1.64)	0.55 (−0.18, 1.28)	0.008
8. Outcome evaluation	0	0.3(0.6)	NA	0.1(0.1)	0.9(1.2)**	1.10 (0.34, 1.87)	NA	NA	NA
9. Continuous quality improvement	0	4.8(4.4)	NA	2.3(2.1)	15.5(6.4)***	2.77 (1.77, 3.77)	NA	NA	NA
10. Sustainability	0	0.0(0.1)	NA	0	0.4(1.1)	NA	NA	NA	NA
Total	8.2(7.9)	34.8(12.6) ***	2.63 (1.67, 3.59)	13.1(13.8)	64.2(26.7)***	2.40 (1.46, 3.34)	0.35 (−0.36, 1.06)	1.33 (0.54, 2.12)***	0.12***

*NA* not applicable because that GTO step was not tested or because there was no variability

*False discovery rate adjusted *p* < .05, significant at the 5 % level

**p < .01, significant at the 1 % level

***p < .001, significant at the 0.1 % level

^a^Hours listed for step 3 are MPC training, the other steps are TA

^b^Tests comparing TA/training hours spent on the intervention vs. control group within year. Greater TA/training hours were spent on the intervention group where noted with asterisks

^c^Tests comparing TA/training hours spent between years 1 and 2 within and between groups. Differences in changes in TA/training hours spent are noted with asterisks

In year 2, the intervention sites had more hours in GTO steps 1 through 9 and total hours than control sites. Including the time it took to deliver the MPC training, intervention sites received about a total 64 h per site compared to about 13 h in the control group. Subtracting the MPC training hours out, it was 34 and 5 h for the intervention and control sites, respectively.

In a comparison of TA hours by year, we found intervention sites had received fewer TA hours in year 2 compared to year 1, *M*_diff_ = −1.3 (95 % CI –1.9 to –0.7), *t*(28) = −4.16, FDR *p* = .001 for step 4. For total TA hours though, intervention sites had received more hours in year 2 compared to year 1, *M*_diff_ = 26.5 (95 % CI 14.8 to 38.2), *t*(28) = 4.64, FDR *p* < .001. In the difference of differences tests, i.e., are the changes between years 2 and 1 different between the two groups, we found that the change in total hours between years 2 and 1 were greater for the intervention group compared to the control group, *M*_diff_ = 22.6 (95 % CI 6.2 to 39.1), *t*(28) = 2.82, FDR *p* = .044. TA hours did not significantly change between years 1 and 2 in the control group.

### Impact of GTO on performance and fidelity

#### Program performance

In year 1, site-level mixed effects regression models indicated intervention sites had significantly greater program performance scores than control sites on the eight GTO steps assessed and the total score (see Table 3 for details, Table 5 for a summary). This means that intervention sites engaged in the various programming practices targeted by GTO with greater quality than control sites. In year 2, intervention sites had greater program performance on seven of the eight GTO steps and the total score compared to the control sites.

**Table 3:** Program performance ratings in years 1 and 2

GTO steps	Year 1^a^			Year 2^a^			Change from year 1 to year 2		
	M(SD)		Hedges’ g (95 % CI)	M(SD)		Hedges’ g (95 % CI)	Hedges’ g (95 % CI)		Generalized omega-squared^b^
	Control	Intervention		Control	Intervention		Control	Intervention	Difference of differences
1. Needs assessment	NA	NA	NA	NA	NA	NA	NA	NA	NA
2. Goals	1.9(0.3)	2.9(0.6)***	2.23 (1.32, 3.14)	1.7(0.5)	3.6(0.8)***	2.95 (1.85, 4.04)	−0.56 (−1.28, 0.16)	1.23 (0.39, 2.07)**	0.20**
3. Best practices	NA	NA	NA	NA	NA	NA	NA	NA	NA
4. Fit	2.1(1.1)	2.9(0.9)*	0.88 (0.14, 1.62)	1.4(0.6)	3.0(1.6)**	1.28 (0.46, 2.09)	−0.72 (−1.44, 0.01)	0.00 (−0.74, 0.74)	0.003
5. Capacity	1.6(0.8)	2.9(1.0)***	1.49 (0.69, 2.28)	1.5(0.6)	3.7(0.9)***	2.97 (1.88, 4.07)	−0.19 (−0.91, 0.53)	1.04 (0.25, 1.83)*	0.077
6. Planning	1.5(0.6)	3.0(1.1)***	1.70 (0.88, 2.52)	1.5(0.7)	3.3(1.4)***	1.62 (0.75, 2.49)	0.01 (−0.71, 0.72)	0.39 (−0.36, 1.14)	−0.013
7. Process evaluation	1.4(0.6)	2.7(0.9)***	1.70 (0.88, 2.52)	2.5(0.9)	3.2(1.2)	0.74 (−0.03, 1.50)	1.75 (0.92, 2.58)*	0.51 (−0.24, 1.27)	0.025
8. Outcome evaluation	1.2(0.8)	2.5(0.8)***	1.73 (0.90, 2.55)	1.1(0.5)	3.2(0.8)***	3.05 (1.96, 4.14)	−0.08 (−0.79, 0.62)	0.88 (0.10, 1.66)*	0.040
9. Continuous quality improvement	1.6(0.8)	2.3(0.7)*	1.00 (0.25, 1.75)	1.6(1.1)	2.6(1.0)*	0.91 (0.13, 1.69)	0.07 (−0.63, 0.78)	0.38 (−0.37, 1.13)	−0.019
10. Sustainability	1.4(0.5)	2.1(0.6)*	0.77 (0.03, 1.52)	2.0(1.2)	3.3(1.2)*	1.10 (0.30, 1.90)	0.62 (−0.10, 1.34)	2.05 (1.12, 2.98)**	0.022
Total	1.6(0.4)	2.7(0.6)***	2.14 (1.25, 3.02)	1.7(0.4)	3.2(0.9)***	2.29 (1.34, 3.25)	0.20 (−0.51, 0.90)	1.33 (0.51, 2.16)*	0.035

Performance ratings were significantly higher for the intervention group where noted with the following asterisks

*NA* not applicable because that GTO step was not tested, *ns* not significant

*False discovery rate adjusted *p* < .05, significant at the 5 % level

**p < .01, significant at the 1 % level

***p < .001, significant at the 0.1 % level

^a^Tests comparing performance ratings between the intervention and control groups within year. Greater performance scores in the intervention group are noted with asterisks

^b^Tests comparing performance ratings between years 1 and 2 within and between groups. Differences in changes in performance ratings are noted with asterisks

Between years 1 and 2, the intervention group significantly improved in performance in steps 2, 5, 8, and 10 and the total score, while the control group significantly improved in performance in step 7 over that time. However, the only significant interaction effect was for step 2, in which the intervention group improved more on performance between years 1 and 2 than the control group, *M*_diff_ = 0.8 (95 % CI 0.4 to 1.3), *t*(24) = 3.76, FDR *p* = .009.

#### Fidelity

Regarding adherence, in year 1, the mixed effects logistic regression model found the intervention group had significantly fewer activities rated as “not at all” compared to the control group (3.8 % of activities vs. 12.3 % respectively, OR 0.35, 95 % CI 0.14 to 0.92, *t*(519) = −2.13, *p* = .033) (see Table 4 for details, Table 5 for a summary). There were no significant differences between groups when comparing activities coded as “fully” vs. [“not at all” + “partially”]. In year 2, because of small cell sizes, we could only model activities rated “fully” vs. [“not at all” + “partially”]. The mixed effects logistic regression models for year 2 found intervention group had significantly more activities rated “fully” (92 %) than the control group (55 %), OR 11.81 95 % CI 4.12 to 33.80, *t*(274) = 4.60, *p* < .001.

**Table 4:** Fidelity comparisons in years 1 and 2

	Year 1					Year 2					Change from year 1 to year 2, odd ratio (95 % CI)			
Adherence: How well was the MPC activity completed?	Control			Intervention		Control			Intervention		Control		Intervention	Difference of differences
	Freq	%		Freq	%	Freq	%		Freq	%	0.97 (0.62, 1.50)		8.65 (4.64, 16.11)***	Logistic *b* = 2.19 (1.43, 2.95)***
Fully	145	55.7		165	57.1	74	55.0		156	92.0^d^				
Partially	83	31.9		113	39.1	48	36.0		12	7.0				
Not at all	32	12.3		11	3.8^c^	12	9.0		1	1.0				
Number of activity observations	260	100		289	100	134	100		169	100				
Quality of delivery (1 = least to 7 = most)^a^	M (SD)				Hedges’ g (95 % CI)	M(SD)				Hedges’ g (95 % CI)	Hedges’ g (95 % CI)			Generalized omega-squared^d^
	Control (*N* = 46)		Intervention (*N* = 51)			Control (*N* = 25)		Intervention (*N* = 29)			Control	Intervention		Difference of differences
Classroom control	4.8 (1.4)		4.7 (1.5)		−0.08 (−0.48, 0.31)	4.7 (1.29)		5.4 (1.10)^e^		0.68 (0.13, 1.23)	−0.13 (−0.62, 0.36)	0.51 (0.05, 0.97)		0.066
Student interest	5.2 (1.4)		5.1 (1.1)		−0.06 (−0.46, 0.34)	4.9 (1.06)		5.6 (0.95)^e^		0.71 (0.16, 1.27)	−0.08 (−0.57, 0.42)	0.89 (0.41, 1.37)		0.051
Facilitator enthusiasm	5.0 (1.1)		4.7 (1.2)		−0.22 (−0.62, 0.18)	4.8 (1.00)		5.5 (0.69)^e^		0.77 (0.21, 1.32)	−0.28 (−0.77, 0.21)	0.39 (−0.07, 0.85)**		0.015**
Objectives met	5.0 (1.3)		5.1 (1.3)		0.09 (−0.32, 0.49)	4.9 (1.07)		6.0 (1.09)^e^		1.18 (0.59, 1.76)	−0.13 (−0.62, 0.36)	0.70 (0.23, 1.17)**		0.058**
Dosage^b^	Control (*N* = 121)		Intervention (*N* = 200)		Hedges’ g (95 % CI)	Control (*N* = 103)		Intervention (*N* = 146)		Hedges’ g (95 % CI)				
Percent modules attended, M (SD)	0.77 (0.24)		0.73 (0.27)		−0.13 (−0.36, 0.09)	0.74 (0.29)		0.69 (0.29)		−0.18 (−0.43, 0.08)	−0.0004 (−0.27, 0.26)	−0.09 (−0.31, 0.12)		−0.025

Differences in changes in fidelity ratings where noted with the following asterisks

*False discovery rate adjusted *p* < .05, significant at the 5 % level

**p < .01, significant at the 1 % level

***p < .001, significant at the 0.1 % level

^a^ *N* = number of modules observed for quality of delivery ratings

^b^ *N* = youth participants with attendance data

^c^In year 1, comparing “not at all” vs. “partially + fully,” OR 0.35, 95 % CI 0.14, 0.92, *t*(519) = 2.13 *p* = .033

^d^In year 2, comparing not at all + partially vs. fully, OR 11.81 95 % CI 4.12, 33.80, *t*(274) = 4.6, *p* < 0.001

^e^In year 2, classroom delivery variables: intervention > control, *t*(51) = 2.49, *p* = 0.016 to *t*(50) = 4.27, *p* < .001

**Table 5:** Study analyses and results summary

Variable	Measures	Significant results
How much GTO was received?		
Implementation support	TA hours delivered in year 1	Intervention group received more TA hours (total and for all GTO steps) than the control group
	TA hours delivered in year 2	Intervention group received more TA hours (total and for all GTO steps) than the control group
	Differences between years 1 and 2	Total TA hours increased for both groups (except for hours spent on GTO step 4), but increased more for the intervention group
What impact did GTO have on sites performance and fidelity?		
Performance	Year 1 interview scores	Intervention group scored higher on performance (total and for all GTO steps) than the control group
	Year 2 interview scores	Intervention group scored higher on performance (total and for all GTO steps except step 7) than the control group
	Differences between year 1 and 2	• Total scores (and scores for steps 2, 5, 8, and 10) increased for both intervention and control groups
		• Step 2 increased more for the intervention group
		• Step 7 increased more for the control group
Fidelity	Year 1 adherence, quality of delivery^1^, and dosage	• Intervention group had fewer activities “not at all” completed
		• No differences between groups on quality of delivery^1^ or attendance
	Year 2 adherence, quality of delivery^1^, and dosage	• Intervention group had more activities completed in full and higher ratings of all quality of delivery variables
		• No differences between groups on attendance
	Differences between year 1 to 2	• Intervention group had more activities rated as completed in full an increase in the quality of delivery (two dimensions: facilitator enthusiasm and objectives met) than in year 1
		• Intervention group had a greater increase in activities rated as completed in full and quality of delivery (two dimensions: facilitator enthusiasm and objectives met) than the control group
Is there empirical support for the GTO logic model?		
GTO predicts performance (intervention group only)	Year 1 TA hours and interview scores	Not significant
	Year 2 TA hours and interview scores	• TA hours predicted performance of GTO step 9 and total score
		• All other GTO steps were not significant
	Change in TA hours and interview scores between year 1 and 2	Not significant
Performance predicts fidelity (both intervention and control group)	Year 1 performance scores and adherence	Higher performance predicts an increase in the odds of an MPC activity being rated “fully” or “partially”
	Year 2 performance scores and adherence	Higher performance predicts an increase in the odds of an MPC activity being rated “fully”
	Differences between year 1 and 2	Not significant

^1^Classroom control, student interest, facilitator enthusiasm, objectives met

Between years 1 and 2, the intervention group had significantly more activities rated “fully” in year 2 than in year 1, OR 8.65, 95 % CI 4.64 to 16.11, *t*(819) = 6.80, *p* < .001. This improvement was greater than the change from year 1 to 2 shown by the control group, OR 0.97, 95 % CI 0.62 to 1.50, *t*(819) = 0.16. The difference between these two groups was significant; the interaction of treatment group and year in our model was significant and positive, logistic *b* = 2.19, 95 % CI 1.43 to 2.95, *t*(819) = 5.64, *p* < .001, indicating those in the intervention group increased their number of activities rated “fully” almost ninefold from years 1 to 2 while in the control group, these ratings remained flat.

Across all four delivery variables, mixed effects regression models showed control and intervention groups did not differ in year 1. In year 2, however, the intervention group had significantly higher ratings for all four delivery variables. In comparing the change in treatment effect by year, facilitator enthusiasm and objectives met improved from year 1 to 2 in the intervention group (classroom control was just beyond significance when adjusted, FDR *p* = .052). Also, the intervention group improved more than the control group from year 1 to 2 on facilitator enthusiasm and objectives met (classroom control, FDR *p* = .057 and student interest, FDR *p* = .057 were both just beyond significance when adjusted).

Regarding dosage, the control and intervention groups did not differ in their percentage of modules attended in years 1 or 2 or between years 1 and 2.

### Support for GTO’s logic model

#### TA hours predicting program performance

From the site-level mixed effects regression models, all steps and total TA hours did not significantly predict program performance in year 1 after correcting for multiple tests. In year 2 within the intervention group, TA hours were associated with a 0.12 increase in step 9 performance (*t*(10) = 3.61, FDR *p* = 0.04). Total TA hours predicted performance, (*t*(10) = 4.83, *p* = .05), but was not statistically significant after correcting for multiple tests (FDR *p* = .16). Within the intervention group, comparing the effect of TA hours on performance by year, there were no significant differences between years 1 and 2.

#### Program performance predicting adherence

In the model testing only performance score across all sites, average performance scores predicted adherence. In year 1, we found a 1-unit increase in performance score was associated with a 2.19 (95 % CI 1.52 to 3.15) increase in the odds of an activity being rated “fully” or “partially” (*t*(519) = 2.17, *p* = 0.031). In year 2, a 1-unit increase in performance score was associated with a 2.29 (95 % CI 1.71 to 3.07) increase in the odds of an activity being rated “fully” (*t*(257) = 2.83, *p* = 0.005) rather than “partially” or “not at all.” The effect of performance score on fidelity (“fully” vs. “partially” + “not at all”) was not significantly different between years 1 and 2.

## Discussion

The EQUIPS study assessed the impact of the GTO implementation support intervention over and above typical EBP training on the performance of key prevention programming practices and fidelity of EBP implementation. In each of the 2 years, BGC sites that received MPC training plus GTO (intervention group) were found to have higher ratings of performance than sites just receiving MPC training (control group). In year 1, this finding was across each of the eight GTO steps rated and the total score. In year 2, all steps were significantly higher for the intervention group except for step 7 (process evaluation) which was close (*p* = .06). By year 2, across most steps, the control group had ratings on the 1–5 scale that were low (1–2 range), whereas the intervention group had moderately high ratings (3–4 range). These findings suggest sites that received GTO demonstrated better performance in areas that GTO targets such as setting goals, ensuring their site appropriately incorporated MPC and had sufficient capacity to carry it out, planning, conducting evaluation, and using data to improve, and planning for sustainability. These are not just GTO steps, but represent important practices that need to be completed well for any EBP [13].

Regarding the adherence dimension of fidelity, in year 1, sites receiving GTO were observed to have fewer instances where they did not carry out an activity of the MPC program at all compared to sites without GTO. However, both groups of sites implemented MPC activities fully only a little more than half the time (55–57 %). In year 2, the intervention group significantly improved their adherence, implementing MPC activities fully 92 % of the time, while the control group remained similar to year 1 (55 %). There was a similar pattern of results between years 1 and 2 for the four classroom delivery variables. None of the four variables (classroom control, facilitator enthusiasm, objectives met, and student interest) were significantly different between the two groups in year 1. However, in year 2, the intervention group had higher ratings on all four variables and significantly improved to a greater extent between years 1 and 2 compared to the control group on two. Dosage (i.e., attendance)—was not different between the groups in either year.

In addition to evaluating GTO impact, we also assessed empirical support for the GTO logic model. Models predicting performance from TA hours (a measure of implementation support) were not significant. It is possible the small sample size made it difficult to detect significant effects (e.g., the model predicting the total performance score from total TA hours was significant before adjustment, approaching significance after adjustment, FDR *p* = .16). Regarding the relationship between performance and adherence, higher total performance predicted greater adherence. The GTO logic model states that organizations demonstrating greater skill in carrying all the various tasks involved in running prevention programs will implement programs with greater fidelity, and these results support that relationship. This relationship is important because it suggests that using the GTO implementation support intervention to increase performance can be effective in improving fidelity, a key factor in achieving positive outcomes from EBPs [26].

Overall, the second year showed more GTO impact and there are several potential reasons. It is possible that the control group did as well in year 1 because they also received support, inadvertently, by their participation in the study. For example, in order to collect fidelity and outcome data, the research staff did need to be in contact with the staff in the control group, which may have organized them in a way similar to what GTO provides (e.g., setting firm dates and locations for program delivery). We also documented intervention bleed as the control group did receive some TA hours, albeit significantly less than the intervention group. In addition to the inadvertent support, the MPC manual and training provide detailed guidance on how to deliver the program. The combination of intervention bleed and MPC structure may have made it difficult to detect many differences in the first year. In the second year, with the addition of GTO’s quality improvement activities, which specifically identified areas of weakness and stimulated plans for improving those areas, the intervention group’s performance and classroom delivery scores were notably higher and adherence scores were nearly perfect.

These findings suggest that in typical community-based settings, manuals and training common to structured EBPs may be sufficient to yield low levels of performance and moderate levels of fidelity, but that more systematic implementation support is needed to achieve high levels of performance and fidelity. We believe these findings are generalizable to low-resourced, community-based settings such as Boys and Girls Clubs. However, we would expect that in organizations with greater resources and more staff, GTO could lead to even better findings. It should be noted that these results were achieved with about 65 h of GTO training and TA time, per site, over the 2-year intervention period. Although more research is needed about the return on investment from implementation support approaches, these findings suggest that using GTO on a large scale could be feasible. For example, the Office of Adolescent Health is funding 75 community-based organizations to implement evidence-based teen pregnancy prevention programs for 5 years. Grantees have been required to use GTO and are receiving TA from OAH staff at the same scope as described here. While it was beyond the scope of EQUIPS, the authors of this report are currently conducting a separate trial, with a very similar design, investigating the cost effectiveness of the GTO support.

Fidelity results in EQUIPS are similar to CTC [51], PROSPER [52], and Rohrbach et al. [32], who documented similarly high rates of adherence or superior fidelity compared to standard training. However, those studies differed in their investigation of what predicted fidelity. For example, PROSPER found some evidence that programs with better adherence had better team meetings, attitudes about prevention, and TA collaboration, although the sample size was small. In contrast, EQUIPS focused on the impact of implementation support and the degree to which improving performance of key programming activities improved fidelity. Future research needs to explore the relative contribution of various factors that can improve fidelity.

There are some limitations that should be noted. First, this report does not present data on youth outcomes. Models of youth outcome data represent separate, though related, research questions that will be addressed in subsequent reports. Second, practitioners at the sites did not have the full experience in doing a needs assessment or searching for and choosing an EBP (GTO steps 1 and 3, respectively). Instead, research staff and club leaders collaboratively decided upon a single EBP (i.e., MPC) prior to the start of the study. This was done to better isolate the impacts of GTO and employ measures of fidelity (and eventually outcomes) that could be similarly compared across all sites. The GTO process was observed—MPC was chosen specifically because data indicated that teen pregnancy rates are higher in the Southern US and among African-American populations, and it is a universal prevention program (i.e., good for all youth) with strong evidence of effectiveness. However, for the purposes of the study, we worked to achieve a consensus across all the sites’ leaders instead of having each site go through the program selection decision on their own. For all other GTO steps, each site individually carried out the related practices. Given the similarity among many universal teen pregnancy prevention programs, we believe GTO received a strong test in EQUIPS. Future studies should be conducted that better isolate the impact of program choice on implementation and outcomes.

Third, we did not evaluate the sustainability of GTO beyond 2 years in this study. While this question needs to be investigated, we believe that community-based practitioners are better able to run programs using the GTO manual on their own (which includes a number of planning and evaluation tools) after receiving the capacity-building support from the GTO technical assistance staff.

Fourth, while we did assess some organizational characteristics at baseline and found no differences between the study groups, it is possible that additional such characteristics that were not measured could have impacted the results. Future GTO studies could benefit from a more comprehensive assessment of such factors. To that end, the current GTO study mentioned above also includes CFIR-based qualitative interviews of representatives at all participating sites that will assess how various factors identified by CFIR impacts the use and effects of GTO.

Finally, although 32 sites are substantial for an RCT, it is a modest sample for evaluating site-level outcomes. Smaller effects may have gone undetected. Future studies are needed in which the impact of implementation support is assessed, using a rigorous randomized design, on a scale that approximates the size of large federally or state funded initiatives such as the CDC’s multi-state efforts [10, 29, 53], or the US Department of Health and Human Services’ Personal Responsibility Education Program [27]. These initiatives are sufficiently large, but their non-experimental designs cannot determine causal impact of implementation support strategies. Only large randomized trials—likely funded by a combination of sources—will be able to shed light on the utility of implementation support at a large scale.

## Conclusions

These findings suggest that sites receiving the GTO implementation support intervention experienced a benefit related to their performance of key programming practices and level of fidelity. Also, this study suggests the quality with which sites perform those practices is related to the level of fidelity those sites achieve, a key link in the GTO logic model. The presence of such a link suggests that GTO (and perhaps other implementation support models like it) not only can help practitioners become more skilled in carrying out programs but also can yield a measurable improvement in the fidelity of specific EBPs. These findings are bolstered by a rigorous design in which we sought to isolate, and then similarly measure, the impacts of GTO over and above the training community-based sites typically receive after acquiring an EBP. Future publications will focus on the degree to which the site-level improvements lead to improved youth outcomes, the final link in the GTO logic model.

## Abbreviations

BGC(s), Boys and Girls Club(s); EBP, evidence-based program; GTO, Getting To Outcomes; MPC, Making Proud Choices; STI, sexually transmitted infection; TA, technical assistance.
